# Brain region-dependent gene networks associated with selective breeding for increased voluntary wheel-running behavior

**DOI:** 10.1371/journal.pone.0201773

**Published:** 2018-08-02

**Authors:** Pan Zhang, Justin S. Rhodes, Theodore Garland, Sam D. Perez, Bruce R. Southey, Sandra L. Rodriguez-Zas

**Affiliations:** 1 Illinois Informatics Institute, University of Illinois at Urbana-Champaign, Urbana, IL, United States of America; 2 Department of Animal Sciences, University of Illinois at Urbana-Champaign, Urbana, IL, United States of America; 3 Beckman Institute for Advanced Science and Technology, Urbana, IL, United States of America; 4 Center for Nutrition, Learning and Memory, University of Illinois at Urbana-Champaign, Urbana, IL, United States of America; 5 Department of Evolution, Ecology, and Organismal Biology, University of California, Riverside, CA, United States of America; 6 Department of Statistics, University of Illinois at Urbana-Champaign, Urbana, IL, United States of America; 7 Carle Woese Institute for Genomic Biology, University of Illinois at Urbana-Champaign, Urbana, IL, United States of America; Technion Israel Institute of Technology, ISRAEL

## Abstract

Mouse lines selectively bred for high voluntary wheel-running behavior are helpful models for uncovering gene networks associated with increased motivation for physical activity and other reward-dependent behaviors. The fact that multiple brain regions are hypothesized to contribute to distinct behavior components necessitates the simultaneous study of these regions. The goals of this study were to identify brain-region dependent and independent gene expression patterns, regulators, and networks associated with increased voluntary wheel-running behavior. The cerebellum and striatum from a high voluntary running line and a non-selected control line were compared. Neuropeptide genes annotated to reward-dependent processes including neuropeptide S receptor 1 (Npsr1), neuropeptide Y (Npy), and proprotein convertase subtilisin/kexin type 9 (Pcsk9), and genes implicated in motor coordination including vitamin D receptor (Vdr) and keratin, type I cytoskeletal 25 (Krt25) were among the genes exhibiting activity line-by-region interaction effects. Genes annotated to the Parkinson pathway presented consistent line patterns, albeit at different orders of magnitude between brain regions, suggesting some parallel events in response to selection for high voluntary activity. The comparison of gene networks between brain regions highlighted genes including transcription factor AP-2-delta (Tfap2d), distal-less homeobox 5 gene (Dlx5) and sine oculis homeobox homolog 3 (Six3) that exhibited line differential expression in one brain region and are associated with reward-dependent behaviors. Transcription factors including En2, Stat6 and Eomes predominated among regulators of genes that differed in expression between lines. Results from the simultaneous study of striatum and cerebellum confirm the necessity to study molecular mechanisms associated with voluntary activity and reward-dependent behaviors in consideration of brain region dependencies.

## Introduction

Reward-dependent behaviors have been linked to learning, memory, and neurological disease processes [1]. High levels of voluntary exercise can be considered a behavior that is dependent on a physical activity reward, and this behavior is also dependent on locomotor control processes [2, 3]. A model based on healthy human subjects proposes that the cortico-striatal and cortico-cerebellar systems contribute differentially to motor sequence learning and motor adaptation, respectively [4].

Mouse lines selectively bred for high voluntary wheel-running behavior have been a helpful model for uncovering the neurological basis of motor learning and adaptation, increased motivation of physical activity, and reward-dependent behaviors [2, 5, 6]. Significant behavioral and physiological differences can be identified between lines selected for high voluntary running and non-selected control lines [2, 3]. For example, differences between high running and control lines in the concentration of monoamines in the substantia nigra pars compacta and dorsolateral striatum, and in the expression of genes coding for chromatin regulators of monoamine receptor in the striatum have been reported [7]. Consistent with the expected association with reward-dependent pathways, we reported differential expression between the striatum of the high running and control lines of genes coding for members of the dopamine signaling pathway, including the neurotransmitters glutamate and GABA and the neuromodulator serotonin [6]. A different set of genes associated with locomotor control, reward-dependent behaviors and dopamine processes, including dopamine receptor D1 and muscarinic acetylcholine receptor M1 were differentially expressed between the cerebellum of the high running and control lines [8]. A more complete understanding of the complementary role of the striatum and cerebellum on the motivation to exercise in particular, and for reward-dependent behaviors in general necessitates the simultaneous analysis of transcriptome between high running and control lines across brain regions.

The goals of this study were: 1) to identify brain-region dependent and independent gene expression patterns and pathways between the high voluntary running and control mouse lines; 2) to integrate information on gene relationships and differential expression between these lines and characterize distinct gene networks between brain regions; and 3) to investigate brain-region dependent and independent differences between the lines in transcription factor regulation. This study advances our understanding regarding the gene networks associated with motivation to exercise that are unique to a brain region or common across brain regions. Understanding the molecular underlining of high voluntary physical activity advances the knowledge on the molecular mechanisms associated with reward dependent and addictive behaviors.

## Materials and methods

### Animal experiments, sampling and sequencing

The experimental procedures were approved by the UCR Institutional Animal Care and Use Committee and were in accordance with the National Institutes of Health Guide for the Care and Use of Laboratory Animals. The cerebellum (Ce) and striatum (St) from 7-week old male mice corresponding to a line selected for high voluntary wheel running (designation line 7) and a control line (designation line 1) were studied. The studied mice were derived from generation 66 of a replicated selective breeding experiment for increased voluntary wheel running behavior on the Hsd:ICR strain [9–11]. Wheel revolutions were recorded in 1-minute intervals, continuously for 6 days and mice were selected within-family for the number of revolutions run on days 5 and 6. In the selected line, the highest running male and female within 10 individual families were selected per generation and each mice was mated to a mice from another family. Mating decisions minimized inbreeding such that the effective population size is approximately 35 [12]. In the control line, one female and one male within each family were selected at random and full sib mating were also prevented [9]. The mice in this study were neither full nor half-sibs. A comparison of the additive genetic variance of 6 locomotor behavior traits between high running and control lines indicated that, albeit the selected line having one third of the variance than the control line, these variances remained fairly constant across 30 generations [13]. By generation 61, mice from four selected lines ran on average approximately 3.3 fold more per day relative to the four control lines [9]. The Ce and St from mice representing the high voluntary running line (Hi) and from the control line (Co) were studied in a 2-by-2 factorial design.

At 35 days old (±5 days) the Hi and Co mice were housed in individual cages with free access to a running wheel and were kept on a 12-hour light/dark cycle with lights on at 0000 h and off at 1200 h for 6 days. Complementary locomotor behavior indicators were measured during the 6 days of the trial including: a) number of wheel revolutions within non-overlapping 30 minute intervals between 150 minutes and up to when the mouse was removed from the cage for dissection; b) maximum number of revolutions per minute in the previously described intervals; c) average number of revolutions per minute in the previously described intervals; and d) average number of revolutions per minute of activity in days 1 to 6.

On day 7 during a period of typical activity, starting at 1300 h and ending at 1700 h (1–4 h after the lights were shut off), mice were removed from their cages with access to running wheel and euthanized by immediate decapitation. Relative to the temporary access to wheel running prior to sampling that all mice in the experiment experienced, the results from the transcriptome comparison between Hi and Co mice are expected to be dominated by the long-term effect of the 66 generations of selection. This expectation is based on a study that compared mice selected for high wheel running versus control and concluded that selection may have affected the motivational system resulting in lower reinforcing value of short running periods on the first group [14].

Brains were extracted, placed on an iced aluminum platform and the entire Ce and St were dissected, immediately transferred to a centrifuge tube and stored at −80°C [15, 16]. Individual brain region samples were homogenized with an RNase-free disposable pellet pestle (Fisher); RNeasy® Lipid Tissue Mini Kit (Qiagen, Valencia, CA) was used for RNA extraction; and DNase I (Qiagen, Valencia, CA) was used to purify the isolated RNA. Qubit® 2.0 (Life Technologies, Carlsbad, CA) was used to assess total RNA yield. The integrity of isolated RNA was measured by the 28S/18S rRNA analysis using the Agilent 2100 Bioanalyzer (Agilent Technologies, Santa Clara CA) and the integrity of the isolated RNA was measured using the Agilent 2100 Bioanalyzer with RNA Pico chip (Agilent Technologies, Palo Alto, CA). The 16 individual samples were evaluated for RNA Integrity Numbers (RIN). Samples were sequenced with the Illumina HiSeq 2000 system (Illumina, San Diego, CA, USA) and 100nt-long paired-end reads from individual mouse and brain regions were generated. The RNA-seq data files are available in the National Center for Biotechnology Information Gene Expression Omnibus (GEO) database (accession identifier GSE114062).

### Differential expression analysis

The analysis compared the transcriptome profiles from 4 groups defined by the brain region followed by the activity line: StHi, StCo, CeHi, and CeCo. These groups encompassed a total of 16 individual mouse samples; half of the samples corresponded of the Hi and the rest to the Co line. Within line; half of the samples corresponded to the St and the rest to the Ce brain region. Quality control of the sequence reads included a minimum average Phred score of 30 across all positions using FastQC [8, 17]. The reads were mapped to the genome assembly *Mus musculus* NCBI GRCm38 using Tophat2 v2.1.1 [18]. The Cuffnorm v2.2.1 routine within the Cufflinks software was used to normalize the gene counts for library size and filter genes inadequately detected across samples [19]. Genes represented in 3 or more sample by one or more read count per million were further studied. The Trimmed Mean of M-values normalized values of 15,213 genes were analyzed for differential expression between activity lines and brain regions. One StHi sample was removed from subsequent testing based on principal component analysis of all genes profiled that suggested deviations from all other samples in the same line-region group.

A linear model including the main effects of activity line, brain region, and the interaction between these effects was used to describe the gene expression patterns. A preliminary analysis of the locomotor measurements indicated that the variation between lines was substantially higher than the variation within lines and thus no additional covariate was included in the model. The analysis was implemented in the edgeR Bioconductor package, v. 3.14.0 within the R v. 3.3.1 software environment [20, 21]. A robust common gene variance was specified using the tag-wise dispersion option to curtail potential false positive results. The quasi-likelihood F-test was used to test for differential expression and the P-values were adjusted for multiple testing using the false discovery rate (FDR) method [22]. Among the possible interaction contrasts, patterns of particular interest were distinct differential gene expression between activity lines within each brain region (StHi–StCo and CeHi–CeCo). These 2 contrasts and StHi-CeHi constitute 3 orthogonal contrasts that can completely characterize the interaction. These results were complemented with a study of the main effect of line by contrasting the gene expression levels between lines (Hi–Co) across brain regions. Likewise, the main effect of brain region was characterized by contrasting the gene expression levels between brain regions (St–Ce).

### Functional, interacting, and regulatory network analyses

Complementary enrichment analyses were used to obtain a comprehensive understanding of the functional categories over-represented among the differentially expressed genes. The categories studied included Gene Ontology (GO) molecular function (MF) and biological process (BP) and Kyoto Encyclopedia of Genes and Genomes (KEGG) pathways [15, 23]. Gene Set Enrichment Analysis (GSEA) considered the expression profiles of all genes analyzed [24]. Genes were ranked based on the signed and standardized logarithm-transformed fold change in the StHi–StCo, CeHi–CeCo, contrasts. The statistical significance of the enrichment was assessed based on P-value from 1,000 permutations and adjusted using FDR. Categories including at least 5 genes were considered. Functional enrichment among genes exhibiting a significant interaction effect and among genes solely exhibiting a significant line effect was performed using the Database for Analysis, Validation, and Integrated Discovery system (DAVID) [25]. EASE scores (modified Fisher Exact) were used to assess the statistical significance of the enrichment of the individual categories [8, 26]. Functional categories including genes in common were clustered and the statistical significance of these functional clusters is represented by the enrichment score (ES) that is the geometric mean of the EASE scores of the categories in the cluster [15, 26]. Further analysis of the enrichment of categories from the Mouse Genome Informatics (MGI) mammalian phenotype database of using Fisher exact test were implemented using the software Enrichr [27].

Gene networks were reconstructed using the BisoGenet package [28] within the Cytoscape environment [29] for the 3 orthogonal contrasts. The edges of the networks depict molecular relationships annotated in the BIOGRID, HPRD, DIP, BIND, INTACT, and MINT databases [30–35] and integrated in the BisoGenet SysBiomics repository. The network framework includes genes that exhibited differential expression in at least one of the 2 orthogonal contrasts between lines (CeCo-CeHi or StCo-StHi) at log2(line fold change) > |1| and P-value < 0.05. This specification ensured adequate connectivity between genes and overlap between brain regions and enabled direct network comparison. The network framework genes are identified by nodes with size representing the fold change magnitude. The remaining network genes connect the framework genes in at least one of the interactome databases but missed the differential expression thresholds used for the framework genes. The comparison of these networks enabled the detection of shared and distinct co-regulation pattern between lines and brain regions.

The study of transcription factor representation among the target genes exhibiting interaction or activity line effects offered further insights into brain region dependent and independent differences in gene regulation. The iRegulon plugin in Cytoscape was used to detect transcription factors, binding motifs, and their optimal sets of direct targets among the differentially expressed genes [29, 36]. Transcription factors that can regulate differentially expressed target genes were identified using the motif position weight matrix [36] and their prevalence was assessed using the normalized enrichment score (NES). Using the iRegulon default parameters, motif sensitivity and specificity was ascribed for minimum receiver operator curve estimation of the area under the curve threshold equal to 0.03 and motif similarity was deemed significant at FDR P-value < 0.001 [36]. Transcription factors supported by > 15% of input genes and that surpassed the default iRegulon NES = 3 cutoff [36] and are discussed.

## Results and discussion

### Locomotor and sequencing statistics

The Se and Co groups were significantly distinct on several locomotor behavior indicators. The Se mice run between 4.4 and 5.5 fold more wheel revolutions during a 30 minute interval (0.0001 < P-value < 0.004) between 150 minutes and before the mouse was removed from the cage for dissection. The Se mice reached between 2.3 and 3.6 fold higher maximum number of revolutions per minute (0.00002 < P-value < 0.002) during the same intervals. The Se mice attained between 3.0 and 4.3 fold higher average number of revolutions per minute (0.0002 < P-value < 0.001) during the same intervals The Se mice attained between 2.7 and 3.4 fold higher average number of revolutions per minute of activity per day (0.01 < P-value < 0.000001) between days 1 and 6 before dissection. These comparisons offer evidence that mice from the Se line ran more and faster than mice from the Co line and that the variation of locomotor indicators between lines is substantially higher than the variation within line.

The number of reads and quality scores along the reads were concordant across samples. The average RIN was consistent across line-brain region groups and ranged from 8.8 to 9.1. The average number of reads per sample was 114,142,993. The average quality score Phred across read positions and samples was 30 indicating a 99.9% base call accuracy and thus reads were not trimmed. The percentage of reads mapped to the mouse genome was consistent across samples and was approximately 92% corresponding to an average of 104,394,396.3 total reads mapped per sample. The normalized values of 15,213 genes were analyzed.

### Voluntary activity line-by-brain region interaction effects on the transcriptome

An innovative comparison of the transcriptome between a line selected for high voluntary physical activity and a control line simultaneously in two brain regions is presented. Table 1 lists the genes that exhibited the most significant line-by-region interaction effects (FDR-adjusted P-value < 0.01, minimum log2 (fold change between lines) > |1.25|). The extended list of 334 genes that exhibited interaction effect (P-value < 0.005) is presented in Table A in S1 File. Among these, 86 genes exhibited a significant interaction effect (FDR-adjusted P-value < 0.05) and the vast majority of these (76 genes) reached the same significance level in at least one line contrast within brain region (StCo-StHi and CeCo-CeHi).

**Table 1:** Genes exhibiting significant (FDR-adjusted P-value < 0.01, minimum log2 (line fold change) > |1.25|) activity line-by-brain region interaction.

Gene		Log2(Line Fold Change)^1^		Line-by-region Interaction	
Symbol	Name	CeCo-CeHi	StCo-StHi	P-value	FDR P-value
Krt25	keratin, type I cytoskeletal 25 isoform X2	-1.98	3.02	1.5E-09	2.3E-05
Lamc2	laminin subunit gamma-2 isoform X2	1.57	-0.59	7.7E-09	5.9E-05
Fam124a	protein FAM124A isoform X1	-1.35	0.03	1.4E-08	7.1E-05
Npsr1	neuropeptide S receptor isoform X2	-6.47	-0.56	4.9E-08	1.9E-04
Myo18b	unconventional myosin-XVIIIb isoform X1	1.40	-1.02	1.4E-07	3.5E-04
Vdr	vitamin D3 receptor	-3.37	-0.39	2.0E-07	4.4E-04
Rab37	ras-related protein Rab-37 isoform 1	0.23	1.61	3.4E-07	6.5E-04
Necab1	N-terminal EF-hand calcium-binding protein 1	-2.69	-0.32	4.2E-07	7.1E-04
Pcsk9	proprotein convertase subtilisin/kexin type 9 precursor	3.21	0.54	1.2E-06	1.8E-03
Tifa	TRAF-interacting protein with FHA domain-containing protein A isoform X1	-1.58	-0.30	6.7E-06	6.0E-03
Creb3l1	cyclic AMP-responsive element-binding protein 3-like protein 1	1.40	0.20	1.0E-05	7.6E-03
Col5a2	collagen alpha-2(V) chain preproprotein	-0.26	1.31	1.2E-05	8.2E-03
En2	homeobox protein engrailed-2	-0.31	1.54	1.3E-05	8.4E-03
Cd300lf	CMRF35-like molecule 1 isoform X3	-1.12	-4.23	1.8E-05	1.1E-02
Npy	pro-neuropeptide Y preproprotein	1.28	-0.11	1.9E-05	1.1E-02

^1^ CeCo-CeHi: cerebellum control versus selected high running line; StCo-StHi: striatum control versus selected high running line.

Further study of the interaction enabled the characterization of the differential expression between activity lines within region among the genes that exhibited a significant interaction effect. Overall, 154 genes exhibited line effect [log2(line fold change > |1|] in each brain region, a difference between regions in the log2(line fold change) > |1|, and interaction effect (P-value < 0.05). Among the 154 genes that exhibited an interaction effect, the majority (107 genes) exhibited differential expression between lines in either or both brain regions (Figure A in S1 File). Furthermore, the Spearman correlation of the log2(line fold change) between both regions based on the 154 genes was -0.034 further confirming that the brain regions studied exhibit distinct associations between gene expression and high voluntary wheel-running behavior.

The expression of various neuropeptide prohormones and related receptor genes exhibited significant line-by-region interaction (Table 1). The over-expression of neuropeptide S receptor 1 (Npsr1) in the Hi relative to Co line in both brain regions, albeit different levels of magnitude, is in line with reports that Neuropeptide S (NPS) and its receptor system have been associated with arousal, anxiolysis, control of fear expression [37], drug addiction, stress [38] and addictive behaviors [39] in human and mouse models. Consistent with the under-expression of Npsr1 in Co relative to Hi observed in this study, mice deficient for Npsr1 did not exhibit NPS-induced hyperlocomotion nor abnormal response to stress [40].

Neuropeptide Y (Npy) was also under-expressed in the Ce of Hi relative to Co mice (Table 1). This neuropeptide modulates reward-dependent and anxiety behaviors. The pattern of Npy gene expression recorded in this study is consistent with reports that decreased concentrations of NPY are implicated in alcohol drinking behaviors and anxiety [41]. Related to neuropeptide production, the proprotein convertase subtilisin/kexin type 9 (Pcsk9) can cleave preprohormones into neuropeptides and biologically active peptides, and in the present study this gene was substantially under-expressed in the Ce of Hi relative to Co line and the same pattern albeit at lower magnitude was observed in the St. In agreement with the over-expression of Pcsk9 in Co relative to Hi mice observed in this study, Pcsk9 was over-expressed in the prefrontal cortex of mice selected for low stress reaction (measured by magnitude of swim stress-induced analgesia) relative to control mice [42].

In our study, vitamin D receptor (Vdr) and N-terminal EF-hand calcium-binding protein 1 (Necab1) showed the same interaction pattern as Npsr1, with over-expression in the Ce of Hi relative to Co mice and to lesser extent in the St (Table 1). Reports of the association between these genes, locomotor regulation, and reward-dependent behaviors support our findings. Vitamin D interacts with the metabolites of several neuropeptides, including oxytocin [43], vasoactive intestinal peptide, and pituitary adenylate cyclase-activating polypeptide [44] that are associated with addiction, reward-dependent, and locomotor behaviors [45]. In agreement with the observed over-expression of Vdr in Hi mice, Vdr-deficient mice have motor impairments that interfere with their ability to float and with post-swimming activity [46]. Necab1 is a candidate marker for commissural interneurons involved in coordinating left/right locomotion and expression in the mouse dorsal root ganglia [47, 48]. Also, the Necab1/2 system serves as a molecular marker for neuron populations of mechanosensory and pain circuits in the spinal cord [48].

Similar to Vdr and Necab1, CD300 molecule like family member F (Cd300lf) was highly over-expressed in the St of Hi relative to Co mice and presented a similar yet four-fold lower differential expression in the Ce (Table 1). This pattern is in agreement with the reported over-expression of Cd3001f in the St of mice that self-administered oxycodone (a highly addictive opioid narcotic) relative to saline controls [49].

Genes associated with reward-dependent behaviors and locomotor control were among those exhibiting highly significant interaction effects and opposite line patterns between the two brain regions (Table 1). Keratin, type I cytoskeletal 25 (Krt25) was over-expressed in Hi relative to Co mice in the Ce and under-expressed in the St. Krt25 was identified as a hub gene in a network of genes associated with Rotarod and Beam Transversal tasks used to assess motor coordination in mice [50], supporting the observed over-expression in the Ce of Hi relative to Co. Reciprocally, homeobox-containing transcription factor engrailed 2 (En2), laminin subunit gamma-2 (Lamc2), and myosin XVIIIb (Myo18b) were all under-expressed in Hi relative to Co mice in the Ce while over-expressed in the St. In agreement with the profiles detected in this study, En2 knockout mice exhibited deficits in specific motor, spatial learning and memory tasks, and altered social behavior including decreased play, reduced social sniffing and allogrooming, and less aggressive behavior [51]. Conforming to our results, Lamc2 was under-expressed in acute nicotine exposure conditions [52] and Myo18b was under-expressed in the midbrain ventral tegmental area that projects to the striatum of rats that self-administered methamphetamine relative to control.

The identification of genes exhibiting significant line-by-brain region interaction effects supports our premise that molecular mechanisms in the Ce and St can have complementary and distinct associations during high voluntary wheel-running behavior. This finding illustrates the additional insights into the molecular basis of the reward-dependent and addictive behaviors offered by the transcriptome comparison of high voluntary physical activity and control mouse lines. Moreover, the annotation of numerous genes exhibiting significant activity-by-region interaction effects to reward-dependent and locomotor control processes endorses the significance of the model and brain regions studied to understand mechanisms associated with addiction-related behaviors.

### Functional analysis of voluntary activity line-by-region interaction effects

The molecular mechanisms that could be impacted by selection for high wheel running on a brain-region dependent manner were detected by functional analysis of the genes exhibiting an interaction effect. Table 2 lists several clusters of enriched (ES > 1.4) GO BP, MF, and KEGG functional categories (> 5 genes exhibiting significant interaction effect and P-value < 0.0001) obtained from the DAVID analysis. A more complete list of clusters (ES > 1.5) is available in Table B in S1 File. The enrichment of the BP nervous system development (GO:0007399) is consistent with reports of similar functional enrichment in the striatum of spontaneously hypertensive rats (SHi) that exhibit hyperactivity behaviors [53, 54], given that lines of mice selected for high wheel running are also hyperactive in home cages when housed without wheels [55]. The over-representation of this category was also observed among genes differentially expressed in the striatum in response to acute morphine [56]. The enrichment of neurogenesis (GO:0022008) is in agreement with reports that running and exercise were able to increase adult neurogenesis in the dorsal dentate gyrus and hippocampus [57, 58]. These categories are consistent with the suggestion that long-term exercise induces plastic changes in the central nervous system, some of which may improve cognition [59].

**Table 2:** Clusters of informative Gene Ontology molecular functions (MF), biological processes (BP), and KEGG pathway categories including at least 5 genes exhibiting an interaction effect and enriched (FDR-adjusted P-value < 0.1) from the DAVID analysis.

Cluster ES /Category	GO Identifier	Name	Count	P-value	FDR P-value
**ES: 2.7**					
BP	GO:0007399	nervous system development	37	1.9E-05	5.0E-02
BP	GO:0022008	neurogenesis	29	5.9E-05	5.3E-02
BP	GO:0007420	brain development	18	7.2E-05	4.8E-02
BP	GO:0031175	neuron projection development	18	8.6E-04	1.3E-01
**ES: 2.1**					
BP	GO:0021695	cerebellar cortex development	5	1.3E-03	1.5E-01
BP	GO:0021587	cerebellum morphogenesis	4	7.0E-03	3.4E-01
BP	GO:0021575	hindbrain morphogenesis	4	8.7E-03	3.7E-01
**ES: 2.0**		** **	** **	** **	** **
BP	GO:0007610	behavior	17	1.1E-04	4.3E-02
BP	GO:0044708	single-organism behavior	11	5.3E-03	3.0E-01
BP	GO:0008344	adult locomotory behavior	5	9.6E-03	3.7E-01
**ES: 2.0**		** **	** **	** **	** **
BP	GO:0007155	cell adhesion	25	1.2E-03	1.5E-01
**ES: 1.5**		** **	** **	** **	** **
BP	GO:0071495	cellular response to endogenous stimulus	22	3.7E-04	8.0E-02
BP	GO:0032870	cellular response to hormone stimulus	12	3.9E-03	2.6E-01
BP	GO:1901653	cellular response to peptide	8	5.3E-03	3.0E-01
**ES: 1.5**		** **	** **	** **	** **
BP	GO:0021953	central nervous system neuron differentiation	7	6.0E-03	3.1E-01
BP	GO:0030900	forebrain development	8	4.4E-02	6.4E-01

The over-representation of cell adhesion (GO:0007155) among the genes exhibiting a significant interaction effect is in agreement with the apparently addictive properties of high voluntary wheel running and is brain-region dependent [60]. Cell adhesion plays a central role in neuronal connectivity and communication, synapse adhesion, and signal transduction, thus providing the fundamental bases for learning, memory, and addiction [61, 62]. The enrichment of BP cerebellum morphogenesis (GO:0021587) and cerebellar cortex development (GO: GO:0021695) is in agreement with its functions in motor control, and in cognitive and motivational processes [8, 63, 64]. The enrichment of the locomotory behavior category (GO:0008344) is consistent with the high voluntary activity line studied, with the role of the cerebellum in locomotor control [65], and is supported by the genes associated with locomotor control that exhibited significant interaction effect. Likewise, the enrichment of the response to hormone and peptide categories (e.g.) is in agreement with reports that wheel-running stimulates endogenous opioids [66, 67] and the anxiolytic effects of voluntary exercise [68]. This finding is supported by the neuropeptide genes (e.g. Nr4a2, Pcsk9, Vdr) exhibiting interaction effect in this study (Table 1).

The significant representation of the GO MF transcription factor (GO:0043565) category (Table B in S1 File) is supported by genes such as the transcription factor En2 (Table 1). This result suggests that regulators of gene expression are associated with high voluntary activity line in a brain region-dependent. The enrichment of a transcription regulatory category is in agreement with reports of a positive association between high wheel running in mice or treadmill running in rats and the expression of transcription factors such as Delta, c-Fos and FosB in the brain [58, 69, 70]. More broadly, this category is in agreement with suggestions that transcription factors contribute to reward-induced adaptations in the brain [71].

Impaired coordination (MP:0001405; P-value < 0.001), increased anxiety-related response (MP:0001363; P-value < 0.001), abnormal cued conditioning behavior (MP:0001454; P-value < 0.001), abnormal locomotor activation (MP:0003313; P-value < 0.003) and hyperactivity (MP:0001399; P-value < 0.01) were the top MGI mammalian phenotype categories enriched among the genes exhibiting interaction effect. Despite not reaching the FDR-adjusted P-value < 0.05 threshold in Enrichr, these phenotypes are aligned with differences in locomotor behaviors between the Se and Co lines [13].

### Functional analysis of voluntary activity lines within brain region

Further insights into the molecular mechanisms potentially underlying the high voluntary activity phenotype on a brain region-dependent manner were gained from the study of functional categories over-represented among genes differentially expressed between lines within a region. Tables 3 and 4 summarize several BP and KEGG categories enriched (FDR-adjusted P-value < 0.05) among the over- and under-expressed genes in the CeCo-CeHi and StCo-StHi contrasts, respectively detected using GSEA. The complete lists of all the terms enriched in both contrasts (FDR-adjusted P-value < 0.2) are available in Tables C and D in S1 File.

**Table 3:** Gene Ontology (GO) biological process (BP) and KEGG pathway categories enriched (FDR-adjusted P-value < 0.05) among the genes over-expressed in the CeCo-CeHi contrast detected using GSEA.

Expression/Category	Identifier	Name	Gene Count	P-value	FDR P-value
**Over-expressed**					
KEGG	mmu00190	oxidative phosphorylation	99	0.0E+00	0.0E+00
KEGG	mmu03010	ribosome	82	0.0E+00	0.0E+00
BP	GO:0045333	cellular respiration	127	0.0E+00	3.7E-04
KEGG	mmu05012	Parkinson’s disease	101	0.0E+00	2.2E-03
KEGG	mmu05016	Huntington’s disease	146	0.0E+00	6.1E-02

**Table 4:** Gene Ontology (GO) biological process (BP) and KEGG pathway categories enriched (FDR-adjusted P-value < 0.05) among the genes over- and under-expressed in the StCo-StHi contrast detected using GSEA.

Expression/Category	Identifier	Name	Gene Count	P-value	FDR P-value
**Over-expressed**					
BP	GO:0006119	oxidative phosphorylation	73	<1.0E-20	2.8E-03
BP	GO:0045333	cellular respiration	129	<0.0E+00	4.2E-03
KEGG	mmu03010	ribosome	82	0.0E+00	5.6E-03
**Under-expressed**					
BP	GO:0010575	+ regulation of vascular endothelial growth factor production	18	0.0E+00	3.6E-02

Consistently enriched functional categories in Ce and St among the genes differentially expressed between Hi and Co mice (Tables 4 and 5) include the KEGG oxidative phosphorylation (mmu00190) and ribosome (mmu03010) pathways, as well as cellular respiration process (GO:0045333). The KEGG Parkinson’s disease pathway was also enriched among the genes over-expressed in StCo-StHi at P-value < 1.0E-10 or FDR-adjusted P-value < 0.15 (Table D in S1 File). Additional GO BPs were enriched in both regions, but these are less specific (e.g. BP cellular respiration) or subsets of the previous three categories (e.g. BP oxidoreductase activity acting on a heme group of donors) and thus further interpretation focuses on the 3 informative categories.

**Table 5:** Top informative genes exhibiting significant (FDR-adjusted P-value < 0.01, log2 (fold change) > |4|) differential expression between Control (Co) and high activity (Se) lines across brain regions.

Symbol	Name	Log2(Co/Hi)	FDR P-value
Zfp33b	low quality protein: zinc finger protein 431-like	-4.46	3.76E-12
Ms4a2	high affinity immunoglobulin epsilon receptor subunit beta isoform c	8.20	4.25E-11
Hist1h2al	histone Cluster 1 H2A Family Member L	5.06	1.26E-09
Arhgap8&	rho GTPase-activating protein 8 isoform X2	-5.13	1.61E-08
Ms4a3	membrane-spanning 4-domains subfamily A member 3	8.16	2.14E-08
Cyp11a1	cholesterol side-chain cleavage enzyme, mitochondrial isoform X1	-5.29	2.82E-08
Lipo2	lipase, member O2 isoform X3	4.30	8.35E-08
Gif	gastric intrinsic factor precursor	8.25	2.02E-05
Krt12	keratin, type I cytoskeletal 12	-4.44	6.49E-05
Shox2	short stature homeobox protein 2 isoform X1	-5.34	0.0092

The over-representation of the oxidative phosphorylation and cellular respiration processes among the genes over-expressed in Co relative to Hi mice (Tables 3 and 4) is consistent with reports that regular exercise decreases the level of reactive oxygen species and modulates protein oxidation in the brain of adult rats [72, 73]. Likewise, the enrichment of the KEGG ribosome pathway among the genes over-expressed in Co relative to Hi mice is consistent with work demonstrating that protein synthesis is noticeably depressed in the brain of swimming rats [74]. The enrichment of Parkinson’s disease in Hi relative to Co is in agreement with a large body of studies in rodent models and humans suggesting that exercise can protects the brain against such neurodegenerative conditions [75–77].

Further study of the consistency of the line expression patterns across regions helped us understand the consistent enrichment of three KEGG pathways (oxidative phosphorylation, ribosome, and Parkinson’s disease) in Ce and St summarized in Tables 3 and 4 despite the non-overlapping differentially expressed genes listed in Table 1. The correlation between regions of the log2(line fold change) of the genes enriching each pathway was computed to assess the consistency of activity line patterns across brain regions. The correlation of gene log2(line fold change) between brain regions for oxidative phosphorylation, ribosome, and Parkinson’s pathways were 0.39, 0.53, and 0.89, respectively. The strong consistency between brain regions of line differences among genes in the Parkinson’s pathway genes is in agreement with reports that exercise augments the level of a dopamine receptor associated with addiction and reward-dependent behaviors in a mouse model of Parkinson’s disease [78]. The brain region-dependent profiles of the genes exhibiting interaction effect yet common enrichment of the Parkinson’s disease pathway observed in this study supports the paradigm that both striatal and cerebellar systems participate in motor skill learning albeit in distinct processes [4]. The striatum processes may predominate during the learning of a sequence of movements that support fast wheel running, whereas the cerebellum processes may predominate during motor adaptation to environmental perturbations such as free access to running wheel. Evidence supporting the previously hypothesized complementary role of these brain systems in motor skill learning has come from impairments found in patients with striatal dysfunction such as Parkinson’s disease [4]. With respect to the other two pathways enriched in genes exhibiting brain-region dependent profiles between lines, ribosome and oxidative phosphorylation pathways were enriched in a rat model of cerebral ischemic tolerance to restricted blood flow [79]. Learning a complicated sequence of voluntary finger movements in humans has been associated with higher regional cerebral blood flow in the cerebellum and that acquisition of the motor skill results in an increase of the regional cerebral blood flow in the striatum [80].

A motivating finding is the enrichment of the BP positive regulation of vascular endothelial growth factor (VEGF) production among genes over-expressed in the St of Hi relative to Co mice (Table 4, Table D in S1 File), whereas no functional category achieved significant enrichment among the genes exhibiting the same pattern in the Ce (Table 3, Table C in S1 File). Despite the presence and role of VEGF in both brain regions, the over-representation of the positive regulation of VEGF in the St of Hi mice is consistent with the neuroprotective effect of VEGF in Parkisons’s disease. Parkinson’s disease patients exhibit dopamine deficit in the St and suffer loss of locomotor control, expressed in rigidity and shaking [81]. These findings lead us to suggest that the high gene expression of VEGF in the Hi mice could support higher locomotor control through the St, rather than the Ce. Our study of Ce and St simultaneously enabled this innovative insight into the molecular mechanisms supporting high voluntary activity.

### Interaction networks of voluntary activity lines within brain region

**Fig 1:**
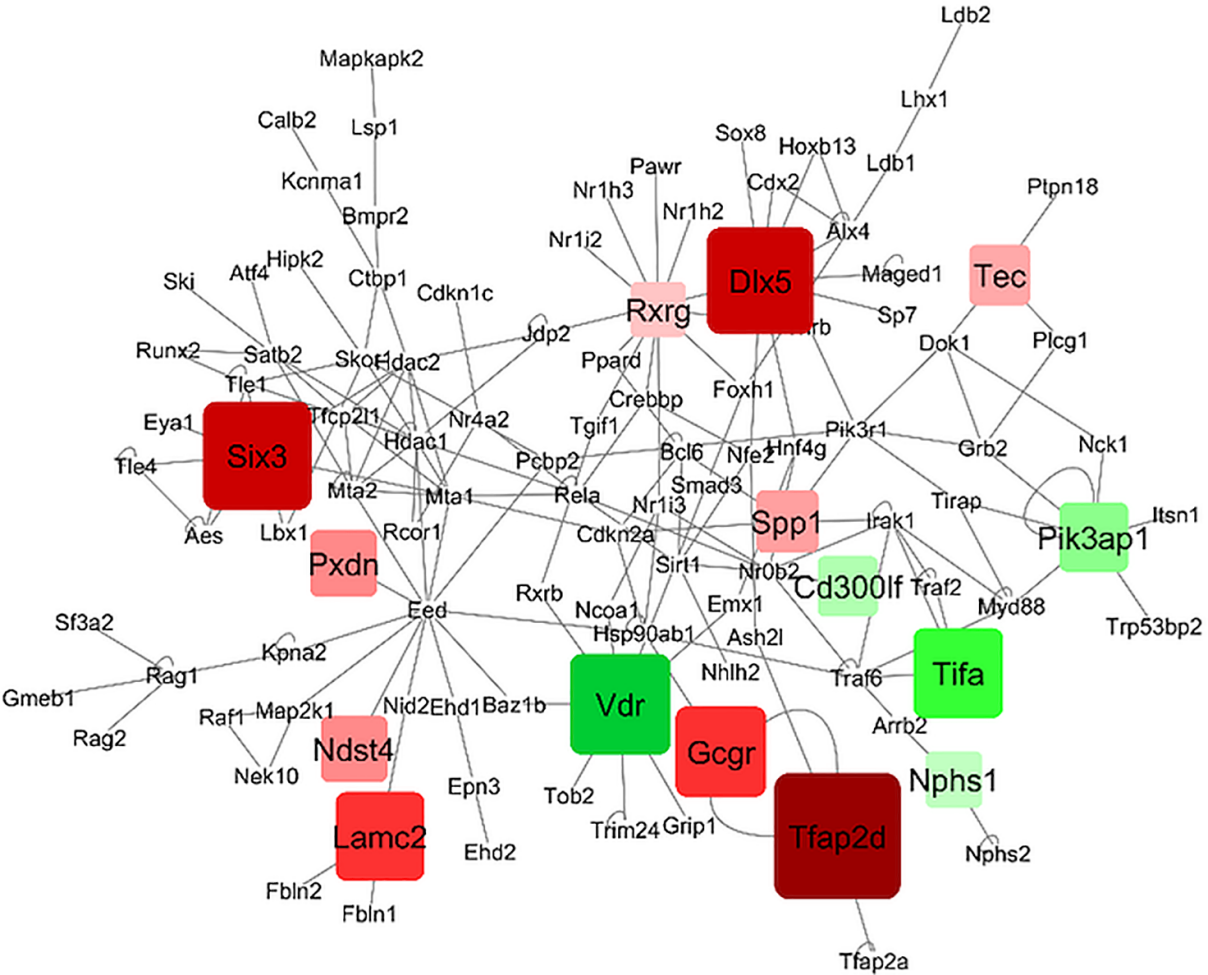
Network of differential gene expression in the cerebellum. Delineated nodes: framework genes that exhibited a line fold change difference between brain regions > |2| and an activity line-by-brain region interaction effect; red: under-expression in Hi and green: over-expression in Hi relative to Co. The size of the node and intensity of the color denote the magnitude of the fold change. Non-delineated nodes: genes that did not reach the differential criteria or connecting genes. Edges: known direct associations between the corresponding nodes.

**Fig 2:**
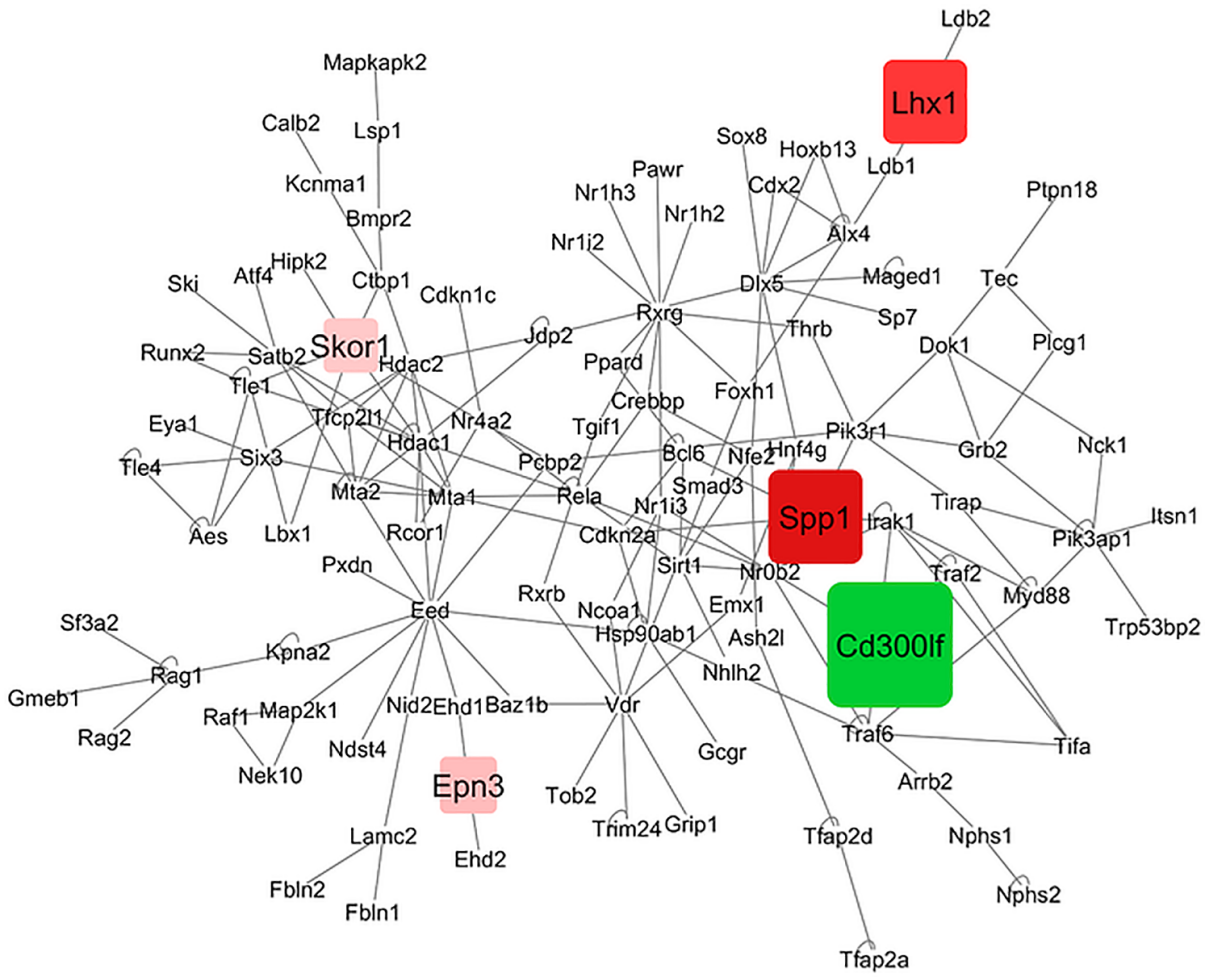
Network of differential gene expression in the striatum. Delineated nodes: framework genes that exhibited a line fold change difference between brain regions > |2| and an activity line-by-brain region interaction effect; red: under-expression in Hi and green: over-expression in Hi relative to Co. The size of the node and intensity of the color denote the magnitude of the fold change. Non-delineated nodes: genes that did not reach the differential criteria or connecting genes. Edges: known direct associations between the corresponding nodes.

Gene networks were inferred to understand the simultaneous impact of line and brain region on multiple gene expression profiles while accounting for known molecular relationships. The reconstructed networks supplemented the discovery of candidate genes and enriched biological processes. Networks were obtained for the contrasts between the Hi and Co lines within brain region. Figs 1 and 2 depict the gene networks for the CeCo-CeHi and StCo-StHi contrasts, respectively. The majority of the genes in the network were differentially abundant at FDR P-value < 0.1 (Six3, Ndst4, Lamc2, Dlx5, Spp1, Vdr, Cd300lf, Tifa, Nphs1, Tfap2d, Tec, Pik3ap1). Four genes differentially abundant at P-value < 0.05 were included to enable connectivity and a more populated network. The network reconstruction centered on a framework of genes that exhibited a line fold change difference between brain regions > |2| and an activity line-by-brain region interaction effect. This strategy enabled the reconstruction of a network of 110 genes, afforded adequate connectivity between framework genes and correspondence between brain regions, and facilitated the interpretation of gene network differences between lines and brain regions. In the networks, green and red rectangular nodes represent over and under-expressed framework genes. The size of the node and intensity of the color denote the magnitude of the fold change. The genes in the network depicted without nodes did not reach the differential expression threshold but connect the framework genes based on the protein-protein interaction databases considered. The edges represent known direct associations between the corresponding nodes.

Two network framework genes, Cd300lf and secreted phosphoprotein 1 (Spp1), reached significant differential expression level in both brain region networks and have been associated with functional categories relevant to this study. Cd300lf was over-expressed whereas Spp1 was under-expressed in Hi relative to Co in both brain regions. Both genes were more differentially expressed in the St than in the Ce. The pattern of these genes in this study is consistent with profiles associated with reward-dependent behaviors. Spp1 is postulated to have neuroprotective activity in the cerebellar cortex in adult rats [82] and was down-regulated in the dorsolateral prefrontal cortex of alcoholic patients relative to controls [83]. Also, this gene is annotated to multiple risk pathways associated with Parkinson’s disease [84]. In our study, the Parkison’s pathway was enriched among the genes differentially expressed between activity lines, and the expression patterns were highly and positively correlated between brain regions. Also, Cd300lf was over-expressed in the striatum of mice that self-administered oxycodone relative to saline controls [49]. Laminin, gamma 2 (Lamc2) was under-expressed in Hi relative to Co mice only in the Ce network. Lamc2 was also down-regulated by acute nicotine exposure [8, 52].

Both networks (Figs 1 and 2) highlight several transcription regulators that are under-expressed in Hi relative to Co and that have been previously associated with reward-dependency studies. In the Ce, these genes include transcription factor AP-2-delta (Tfap2d), Sine oculis homeobox homolog 3 (Six3), and distal-less homeobox 5 gene (Dlx5). Tfap2d is a key transcriptional regulator of the posterior midbrain region that adjoins the cerebellum [85] and was identified among the genes associated with preferential amplification in the dorsal dentate gyrus of rats administered methamphetamine relative to saline [86]. Six3 is a transcription regulator that has been associated with alcohol and nicotine co-dependence [87]. In a study of Lewis rats known to be more vulnerable to drugs of addiction than Fischer 344 rats, Dlx5 exhibited differential expression in the nucleus accumbens and frontal cortex between both strains [88]. Dlx5 was proposed to activate transcriptional networks required for GABAergic fate specification of neurons in the forebrain [89].

In the St network, epsin 3 (Epn3) and the transcription regulators LIM/homeobox protein Lhx1 (Lhx1) and SKI family transcriptional corepressor 1 (Skor1) were under-expressed in Hi relative to Co mice. Consistent with our results, Lhx1 was over-expressed in the striatum of rats treated with the sedative Isoliquiritigenin that reduced cocaine-triggered locomotor activity [90]. Variants in Skor1 have been associated with restless leg syndrome that has high incidence during opioid withdrawal periods [91]. The comparison of the gene networks of differential expression between activity lines across brain regions exemplified the mostly distinct impact of high voluntary activity in the Ce and St.

### Brain-region independent effect of activity line

Genes that exhibited differential expression between activity lines independent of brain region (excluding genes with a significant interaction effect) were studied. Table 6 lists several top differentially expressed genes between activity lines (FDR-adjusted P-value < 0.01, log2(line fold change) > |4|). An extended list of 144 genes log2(Co/Se) > |1| is presented in Table E in S1 File.

**Table 6:** Lists top differentially expressed genes between the cerebellum (Ce) and the striatum (St) at FDR-adjusted P-value < 1.0 E-19 and log2(fold change) > |5|.

Symbol	Name	log2(Ce/St)	P-value	FDR P-value
Homer3	homer protein homolog 3 isoform X1	5.47	4.7E-24	3.6E-20
Car8	carbonic anhydrase-related protein	7.09	4.7E-24	3.6E-20
Cbln3	cerebellin-3 precursor	10.79	1.1E-23	5.5E-20
Gabra6	gamma-aminobutyric acid receptor subunit alpha-6 isoform X1	11.52	7.9E-23	2.4E-19
Atp2a3	sarcoplasmic/endoplasmic reticulum calcium ATPase 3 isoform a	5.67	7.3E-22	1.2E-18
Foxg1	forkhead box protein G1	-9.40	7.6E-22	1.2E-18
Pcp2	Purkinje cell protein 2 isoform X2	10.48	2.1E-21	2.3E-18
Rin1	ras and Rab interactor 1 isoform X4	-5.09	4.0E-21	3.3E-18
Adora2a	adenosine receptor A2a isoform X2	-7.40	4.8E-21	3.7E-18
Fat2	protocadherin Fat 2 isoform X2	11.20	5.2E-21	3.7E-18
Arhgef33	rho guanine nucleotide exchange factor 33 isoform X1	7.38	5.4E-21	3.7E-18
Chrm4	muscarinic acetylcholine receptor M4	-5.93	6.4E-21	3.9E-18
Cbln1	cerebellin-1 precursor	6.40	1.9E-20	9.3E-18
Dbpht2	DNA binding protein with his-thr domain	-5.21	2.0E-20	9.4E-18

Many of these genes have been associated with reward-dependent behaviors and with locomotor regulation in a manner similar to that observed in the present study. Among the genes in Table 5, cytochrome P450 11A1 (Cyp11A1) was over-expressed in the Hi relative to the Co mice in both brain regions. This gene modulates the brain sensory and motor systems and locomotor coordination through the involvement of the coded enzyme in the modifications of neurosteroidogenesis-related proteins and neurosteroids [92].

The differential expression of rho GTPase activating protein 8 (Arhgap8) in Hi relative to Co mice is consistent with reports that several rhoGAPs are coded by a gene that modulates stimulant and sedative behaviors, such as motor incoordination induced by ethanol in the fruit fly [93]. The over-expression of keratin, type I cytoskeletal 12 (Krt12) in Hi relative to Co mice is consistent with the detected association between mutations in this gene and sensitivity of mice to the locomotor stimulant methamphetamine [94]. Likewise, the over-expression of Shox2 in the Hi relative to Co mice is consistent with the association between the expression of this gene and chronic substance abuse treatments in mice [95].

The functional enrichment analysis of the 122 genes differentially expressed between Co and Hi mice was implemented in DAVID because of the removal from consideration of genes exhibiting an interaction effect. Among these genes, 24 genes were annotated to the GO BP immune system process (GO:0002376), 16 genes were annotated to the GO BP immune response (GO:0006955), and 7 genes to the KEGG pathway cytokine-cytokine receptor interaction (mmu04060). These categories were enriched at P-value < 0.0007) yet did not reach the FDR-adjusted P-value < 0.1. The enrichment of MGI mammalian phenotype categories including: abnormal immune system organ morphology (MP:0002722; P-value < 0.0008), decreased tumor necrosis factor secretion (MP:0008561; P-value 0.003) detected by EnrichR among the differentially expressed genes support the previous findings. Overall, a limited number of genes exhibited differential expression between lines exempt of interaction effect, and the known function of several of these genes support the categories identified for genes exhibiting line-by-brain region interaction effects.

### Main effect of brain region on gene expression

The focus of this study was to further the understanding of the transcriptome differences between a high voluntary activity relative to control line in both or either the St and Ce. The comparison of gene expression between brain regions presented here serves as molecular confirmation of the regions studied and analytical completeness. The comparison of brain regions also offers evidence of the statistical power of the experimental design used to detect genes known to be differentially expressed between brain regions based on the mouse ENCODE transcriptome database [96]. Supporting the expected differences between regions, 243 genes presented a significant (FDR-adjusted P-value < 1.0E-15) brain region effect, excluding genes exhibiting an interaction effect. Table 6 lists the top differentially expressed genes (FDR-adjusted P-value < 1.0 E-19, log2(region fold change) > |5|) and an extended list of genes is presented in Table G in S1 File.

Several known molecular markers of Ce and St were differentially expressed in this study, confirming the representativeness of the brain region samples profiled. Consistent with known expression patterns of genes for the 2 brain region studied in the mouse ENCODE database, Homer3, Car8, Cbln3, Gabra6, Pcp2, Fat2, Arhgef33, and Cbln1 are uniquely or highly over-expressed in the Ce relative to the whole brain whereas Foxg1, Rin1, Dlx6as1, Chrm4, and Dlx1as are highly over-expressed in non-cerebrum regions relative to the Ce.

### Regulatory networks of brain-region dependent and independent activity line effects

The Ce and St gene networks in Figs 1 and 2 highlight the differential expression between activity lines of genes that regulate the transcription of other genes. This finding was corroborated by the enrichment of MF category related to transcription regulation among the genes that exhibited an interaction effect. Further understanding of this phenomenon was gained by studying the enrichment of transcription factors among the 276 potential target genes that exhibited interaction and activity line main effects. Both groups of genes were analyzed together for potential co-regulatory enrichment because transcription factors may impact the expression of some target genes on a brain region-dependent and on a brain-independent manner in other genes.

**Fig 3:**
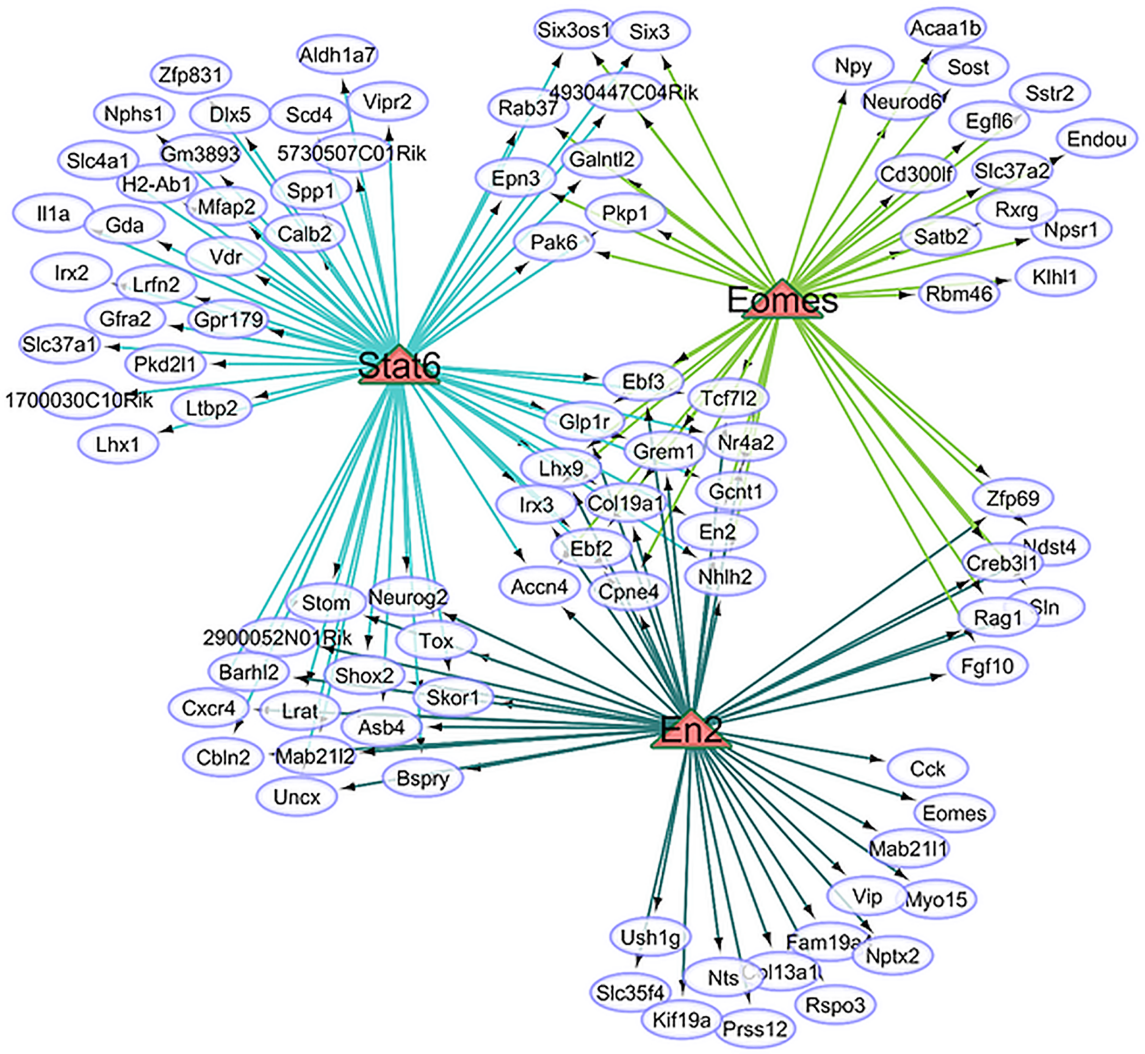
Transcription factors enriched among the genes exhibiting line-by-region interaction or activity line main effect. Triangles: transcription factors; ovals: target genes.

Table 7 summarizes the transcription factors over-represented (NES > 3.5, > 15% of all the differentially expressed genes analyzed) together with the number of target genes, number of motifs used to assign the transcription factors to the genes, and the magnitude of the interaction and line effects on the transcription factor. Fig 3 depicts the relationships between the 3 transcription factors (triangles), target genes (ovals) and the targets of multiple transcription factors.

**Table 7:** Transcription factors over-represented (normalized enrichment score > 3.5, targeting > 15% of all the differentially expressed genes considered) among genes exhibiting interaction and line effect and identified by at least 2 motifs.

Transcription factor	NES^1^	Target Count^2^	%^3^	Motif/Track Count^4^	Interaction Effect		Line Effect		
					P-value	FDR P-value	Log2(Co/Se)	P-value	FDR P -value
Stat6	3.80	61	22.1	4	3.5E-01	8.5E-01	0.21	3.2E-02	1.7E-01
En2	3.49	48	17.4	12	1.3E-05	8.4E-03	-0.62	7.7E-04	1.6E-02
Eomes	3.49	42	15.2	2	1.3E-01	6.9E-01	-1.10	1.6E-03	2.5E-02

^1^NES: normalized enrichment score or maximal enrichment score for a transcription factor

^2^ Number of target genes

^3^ Percentage of potential target genes out of all genes analyzed

^4^ Number of motifs per transcription factor

The 3 transcription factors most enriched were: En2, signal transducer and activator of transcription 6 (Stat6), and eomesodermin (Eomes), and these transcription factors have been associated with behavior, locomotor, and dopamine processes. En2 exhibited a significant interaction effect characterized by under-expression in the St of Hi relative to Co mice (Table 1). This trend is consistent with reports that En2-deficient mice perform under-par in motor, spatial learning and memory tasks [51]. Eomes is highly expressed in the cerebellum compared to the frontal cortex and is associated with neurodevelopment [97]. Stat6 was under-expressed in Hi relative to Co mice and this pattern is consistent with reports that Stat6-deficient mice exhibited lower immobility time in the forced swimming test relative to wild type mice [98].

Fig 3 depicts the association between En2 and Eomes through a reciprocal regulation of each other and this mode of action is in agreement with the consistent over-expression of both genes in Hi relative to Co mice (Table 5). En2 and Eomes have been associated with multiple neuropeptide genes that exhibited line-by-region effects including vasoactive intestinal polypeptide (Vip), neurotensin (Nts), cholecystokinin (Cck), neuropeptide Y (Npy), and the receptor Npsr. This finding indicates that members of the neuropeptidome system have distinct profiles between activity lines. Moreover, the association between neuropeptides and activity line are distinct between the Ce and the St.

## Conclusions

The innovative comparison of the transcriptome between two mouse lines that exhibit different levels of voluntary wheel-running behavior simultaneously in the Cerebellum and Striatum offered novel insights into brain-region specific and common differential gene expression and networks that can be associated with reward-dependent behaviors. The analysis of locomotor measurements between days 1 and 6 prior to dissection indicated that the mice from the selected line run substantially further and faster than the mice from the control line. The ratio between selected and control mice on average number of revolutions per minute of activity per day remained fairly uniform between days 1 and 6 prior to dissection. This result does not offer evidence of differential sensitization to running wheel access for the mice and period studied although sensitization during longer trials should be assessed. The majority of these genes were differentially expressed between activity lines in either one or the other brain region, with approximately even distribution between Ce and St.

The differentially expressed genes detected in this study have been previously associated with reward-dependent behaviors, locomotor control, and neurological or behavioral disorders. Moreover, the differential expression patterns discussed were consistent with patterns observed in relevant studies of addiction, locomotor behaviors, and/or neurological disorders. Insufficient resources prevented further validation of the profiles using different quantitative technologies. On the other hand, the detection of differential expression of multiple genes that are accepted molecular markers of the corresponding brain regions from other studies supports the capacity of the experimental design to detect molecular differences.

Applying the environmental conditions of the high voluntary running and control lines, all mice had access to running wheels prior to sampling. This practice minimized the likelihood that highly motivated mice would experience stress akin to withdrawal from reward if blocked from wheel access. On the other hand, the used practice hinders the possibility to distinguish the effect of a genetic basis of motivation for wheel running from the effect of running per se on the transcriptome. Further studies comparing female and male mice enabled or blocked from physical activity can offer insights into potential multi-factorial interactions impacting gene expression profiles.

A breakthrough result was the identification of 3 KEGG pathways that were enriched among the genes differentially expressed between activity lines in both brain regions studied. Among these, the pattern of expression between lines of genes in the enriched Parkinson’s pathway was high and positively correlated between regions whereas the patterns in the enriched oxidative phosphorylation and ribosome pathways were less consistent between brain regions. This result highlights how the same selection for high voluntary activity can impact some molecular mechanisms in a consistent manner across brain regions while other processes are impacted in distinct manner across regions. The simultaneous study of St and Ce enabled us to confirm the predominant brain-region dependent association of transcription factors with activity line through their target genes. Our study of transcriptional regulation advanced the understanding of the inherent role of transcription factors in modulating voluntary exercise and possibly reward-dependent behaviors in general.

A compelling finding was the prevailing differential expression of genes in the neuropeptide system between lines across the brain regions studied. Moreover, the enriched transcription factors detected in this study are associated with neuropeptide and related genes. The predominant brain-region dependent differential gene expression associated with lines that exhibit distinct voluntary activity behavior supports the necessity to develop biomarkers and therapies for reward-dependent behaviors in consideration of brain-region dependencies.

## Supporting information

S1 File Extended lists of differentially expressed genes and enriched functional categories. Table A. Extended list of genes exhibiting activity line by brain region interaction effect.
Table B. Extended list of clusters of Gene Ontology molecular functions, biological processes, and KEGG pathways enriched among genes exhibiting activity line-by-brain region using DAVID.
Table C. Extended list of enriched Gene Ontology (GO) terms among genes differentially expressed between activity lines selected and Control mice in the cerebellum using GSEA.
Table D. Extended list of enriched Gene Ontology (GO) terms among genes differentially expressed between activity lines selected and Control mice in the striatum using GSEA.
Table E. Extended list of genes exhibiting activity line main effect.
Table F. Extended list of enriched Gene Ontology (GO) terms among genes exhibiting activity line main effect using GSEA.
Table G. Extended list of genes exhibiting brain region main effect.
Figure A. Venn diagram of number of genes that exhibited line effect within each brain region and activity line-by-interaction effect.
(XLSX)
